# Transcriptional Control in the Segmentation Gene Network of *Drosophila*

**DOI:** 10.1371/journal.pbio.0020271

**Published:** 2004-08-31

**Authors:** Mark D Schroeder, Michael Pearce, John Fak, HongQing Fan, Ulrich Unnerstall, Eldon Emberly, Nikolaus Rajewsky, Eric D Siggia, Ulrike Gaul

**Affiliations:** **1**Laboratory of Developmental Neurogenetics, Rockefeller UniversityNew York, New York, United States of America; **2**Center for Studies in Physics and Biology, Rockefeller UniversityNew York, New YorkUnited States of America

## Abstract

The segmentation gene network of *Drosophila* consists of maternal and zygotic factors that generate, by transcriptional (cross-) regulation, expression patterns of increasing complexity along the anterior-posterior axis of the embryo. Using known binding site information for maternal and zygotic gap transcription factors, the computer algorithm Ahab recovers known segmentation control elements (modules) with excellent success and predicts many novel modules within the network and genome-wide. We show that novel module predictions are highly enriched in the network and typically clustered proximal to the promoter, not only upstream, but also in intronic space and downstream. When placed upstream of a reporter gene, they consistently drive patterned blastoderm expression, in most cases faithfully producing one or more pattern elements of the endogenous gene. Moreover, we demonstrate for the entire set of known and newly validated modules that Ahab's prediction of binding sites correlates well with the expression patterns produced by the modules, revealing basic rules governing their composition. Specifically, we show that maternal factors consistently act as activators and that gap factors act as repressors, except for the bimodal factor Hunchback. Our data suggest a simple context-dependent rule for its switch from repressive to activating function. Overall, the composition of modules appears well fitted to the spatiotemporal distribution of their positive and negative input factors. Finally, by comparing Ahab predictions with different categories of transcription factor input, we confirm the global regulatory structure of the segmentation gene network, but find *odd skipped* behaving like a primary pair-rule gene. The study expands our knowledge of the segmentation gene network by increasing the number of experimentally tested modules by 50%. For the first time, the entire set of validated modules is analyzed for binding site composition under a uniform set of criteria, permitting the definition of basic composition rules. The study demonstrates that computational methods are a powerful complement to experimental approaches in the analysis of transcription networks.

## Abstract 2

Starting with known transcription binding site information these researchers use a the computer algorithm, Ahab, to recover known control elements and find novel modules within the genome

## Introduction

The development of higher eukaryotes depends on the establishment of complex spatiotemporal patterns of gene expression. Thus, an important key to understanding development is to decode the transcriptional control of patterned gene expression.

**Figure 6:**
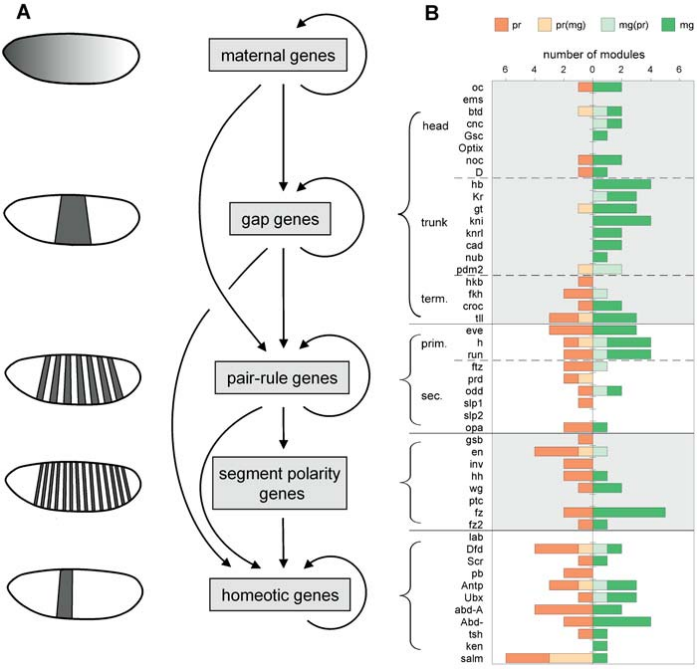
Module Predictions within the Segmentation Gene Network (A) Schematic depiction of the regulatory relationships within the segmentation gene network. (B) Ahab-predicted modules in the control regions of segmentation genes were classified based on their composition into pair-rule driven (pr, red), maternal/gap driven (mg, green), and mixed but predominantly pair-rule (pr(mg), light red) or predominantly maternal/gap driven (mg(pr), light green); see text for details. For each gene, the number and type of modules in the control region is shown; grouping of genes is indicated by brackets and follows the hierarchy as depicted in (A). The type of regulatory input a gene receives is indicative of its position within the gene network.

The segmentation gene network of *Drosophila* has long been one of the prime paradigms for studying the role of transcription control in pattern formation (Carroll 1990; Rivera-Pomar and Jackle 1996). The regulation within the network is almost entirely transcriptional, and many of the *cis*- and *trans*-acting components are well characterized. The network comprises maternal and zygotic factors that act in a hierarchical fashion to generate increasingly refined and complex expression patterns along the anterior-posterior (ap) axis in the blastoderm embryo (St Johnston and Nusslein-Volhard 1992; Driever 1993; Pankratz and Jäckle 1993; Sprenger and Nüsslein-Volhard 1993; St Johnston 1993; Furriols and Casanova 2003): The maternal factors form gradients stretching along the entire ap axis; the zygotic gap factors are expressed in one or more broad, slightly overlapping domains; the pair-rule genes are expressed in seven stripes and segment-polarity genes in fourteen stripes, prefiguring the segmental organization of the larva; finally, the homeotic genes specify segment identity (for schematic see Figure 6A).

Many of the segmentation genes are transcription factors themselves; their principal targets are segmentation genes acting at the same level or below. From a large body of genetic and molecular studies (for review see Akam 1987; Cohen and Jurgens 1991; McGinnis and Krumlauf 1992; St Johnston and Nusslein-Volhard 1992; Martinez Arias 1993; Pankratz and Jäckle 1993), the following broad rules for regulation within the network have been gleaned (cf. schematic in Figure 6A): Gap genes receive input from the maternal factors; the gap genes of the trunk heavily cross-regulate, while the gap genes of the head do not. The pair-rule genes are divided into a primary and a secondary tier: The primary pair-rule genes generate their seven-stripe pattern mainly through maternal and gap input, while the secondary pair-rule genes depend on (primary) pair-rule gene input; but the debate about which pair-rule genes belong to the primary tier is not resolved (Carroll 1990; Klingler and Gergen 1993; Klingler et al. 1996). Segment-polarity genes receive pair-rule gene input, and the homeotic genes receive both gap and pair-rule input.

Like other factors controlling the transcription of protein-encoding genes, the segmentation gene transcription factors bind to *cis*-regulatory elements, also called modules, and positively or negatively regulate the recruitment of the basal transcription machinery to the core promoter (for review see Gray and Levine 1996; Arnone and Davidson 1997; Zhou et al. 1997; Blackwood and Kadonaga 1998; Roeder 1998; Naar et al. 2001; Roth et al. 2001; Arnosti 2003). Specifically, the maternal factors were found to act as activators, while the gap factors act mostly as repressors; however, there is a body of data suggesting that gap factors can act as activators or repressors in a context-dependent fashion (see below).

The expression patterns of the segmentation genes are typically complex, and in many cases different aspects of the pattern are controlled by separate modules. An individual module typically receives input from multiple transcription factors and contains multiple binding sites for each of the factors; in many cases the relevant binding sites are clustered within a small interval of 0.5–1 kb. The combinatorial and redundant nature of the input and its clustering are features that are readily exploited for the computational detection of transcriptional control elements.

We have recently developed an algorithm, Ahab, which uses a thermodynamic model to detect *cis*-regulatory modules (Rajewsky et al. 2002). Ahab uses binding site information for multiple transcription factors participating in a common process and seeks an optimal binding of the factors to a given sequence window. Binding site information for the factors is provided in the form of position weight matrices (Stormo 2000), which Ahab uses to infer binding energies. Ahab then optimizes the total free energy of binding the factors to the sequence. The factors compete for binding with a local background model computed from the base composition within the sequence window; the competition between factors is treated as in standard thermodynamics. The result is then the best partitioning of the sequence window into binding sites and background. The total free energy under this partitioning is taken as the score, and can be used to rank modules. Thus, in contrast to other methods for module detection (Berman et al. 2002; Halfon et al. 2002; Markstein et al. 2002; Papatsenko et al. 2002; Rebeiz et al. 2002), Ahab requires no predefined factor-dependent cutoffs, which means that clusters of weak sites can be detected. We used Ahab for a genome-wide prediction of segmentation gene modules with maternal and gap input and found that it recovers known modules with excellent success (Rajewsky et al. 2002).

Here, we use Ahab to identify novel modules within the segmentation gene network. We test 16 significant novel predictions and find that 13 faithfully produce pattern elements of the endogenous gene, while the remaining three produce more or less aberrant blastoderm patterns. Our combined computational and experimental analysis increases the number of characterized segmentation modules by 50% and provides effective de novo control region dissections for ten of the 29 genes with gap and pair-rule patterns. Furthermore, we systematically analyze Ahab's prediction of binding site composition for all experimentally validated modules. By correlating the expression patterns of modules with their binding site composition, we are able to show that the composition of modules is generally well fitted to the distribution of input factors, and we are able to determine the mode of action for six of the nine maternal/gap input factors. Finally, we explore Ahab's predictive ability when binding site information is less well defined, as is the case with the pair-rule factors. Despite the handicap, Ahab traces the global architecture of the segmentation gene network and pinpoints the unexpected behavior of *odd skipped* as a primary pair-rule gene.

## Results

### Prediction and Validation of Segmentation Modules

As the principal arena for our investigation, we selected the top two tiers of the segmentation gene network, namely the gap and pair-rule genes (for references see Dataset S1). Using Ahab, we searched the genomic regions surrounding these genes for *cis-*regulatory modules containing clusters of binding sites for maternal and gap factors.

**Figure 1:**
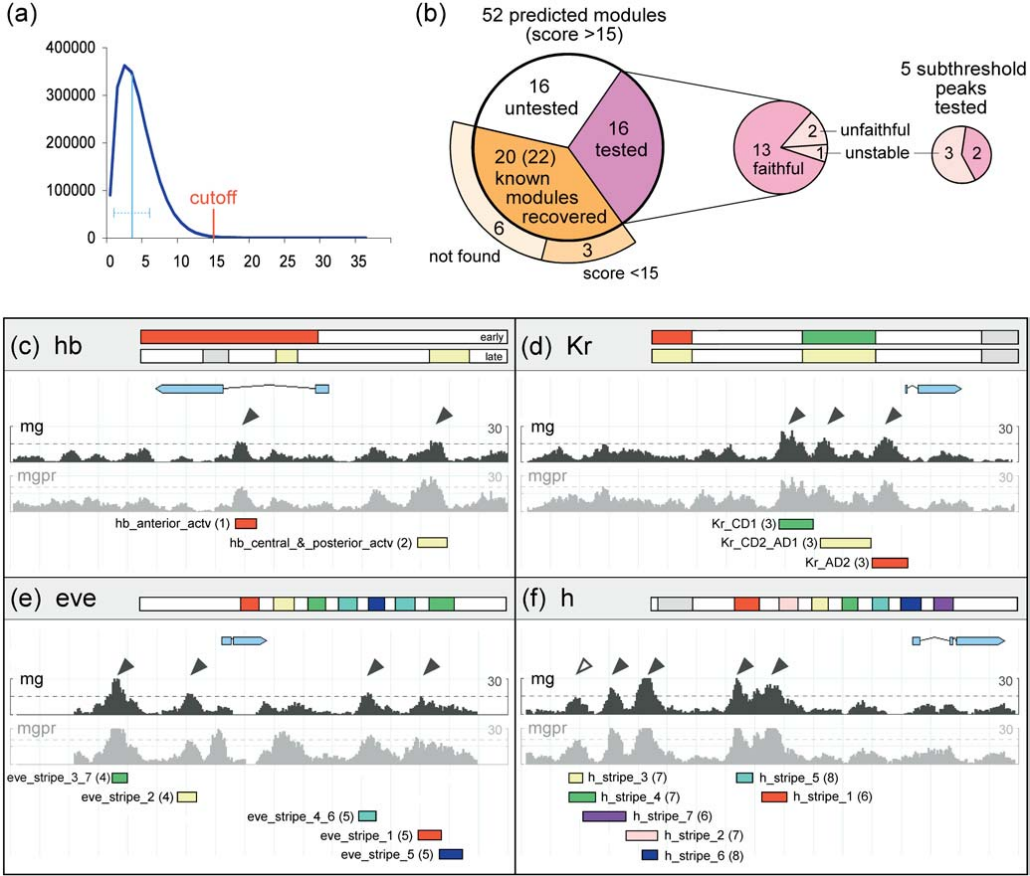
Ahab Predictions and Recovery of Known Modules (A) Histogram of genome-wide window scores for the Ahab mg run (maternal/gap input, window size 500 bp, window shift 50 bp, background model 2). As free energy cutoff we chose 15, which is approximately four standard deviations above the genome-wide mean (indicated by light blue line). (B) Pie chart summarizing results of Ahab predictions for gap and pair-rule genes, including recovery of known modules and testing of novel predictions. (C–F) For the genomic regions of selected gap and pair-rule genes, the free energy profiles of two Ahab runs (mg and mgpr) are shown. The free energy cutoffs are marked by dotted lines; statistically significant predictions for the mg run are marked by black arrow heads (cf. Figure 4). In the header above, the blastoderm expression pattern of the locus is depicted schematically, anterior to the left, posterior to the right. The position of experimentally validated modules within the control region is delineated by colored bars; the aspect of the endogenous pattern they drive is highlighted in matching color. Overall, the control regions of the gap genes *hb* and *Kr* and of the primary pair-rule genes *eve* and *h* are computationally well delineated with maternal/gap input. References: (1) Schroder et al. (1988), (2) Margolis et al. (1995), (3) Hoch et al. (1990), (4) Goto et al. (1989), (5) Fujioka et al. (1999), (6) Riddihough and Ish-Horowicz (1991), (7) Howard and Struhl (1990), and (8) Langeland and Carroll (1993).

As input for Ahab, we provided binding site information (in the form of position weight matrices derived from the literature; Dataset S2) for nine transcription factors: the maternal factors Bicoid (Bcd), Hunchback (Hb), Caudal (Cad), the Torso-response element (TorRE), and Stat92E (D-Stat), and the gap factors Kruppel (Kr), Knirps (Kni), Giant (Gt), and Tailless (Tll). Note that the weight matrices for Kni and Tll are quite unspecific, which leads to an increased number of binding site predictions. Conversely, the available binding site information for D-Stat and Gt is rather limited and thus appears artificially specific, resulting in fewer predictions. Ahab was run over the genomic regions of 29 genes with gap and pair-rule patterns consisting of 0.75 Mb of total genomic sequence (see Materials and Methods). We experimented with two adjustable parameters of Ahab, free energy cutoff and the order of the background model, i.e., whether pairs or triples of bases are used as background sequence. We favored the lower order background, which is less stringent and increases the number of factor binding sites, and set the free energy cutoff at 15, which is approximately four standard deviations above the mean of genome-wide window scores (Figure 1A). The window size was set at 500 bp, which we had previously found to deliver the most efficient recovery of known modules (Rajewsky et al. 2002).

**Figure 4:**
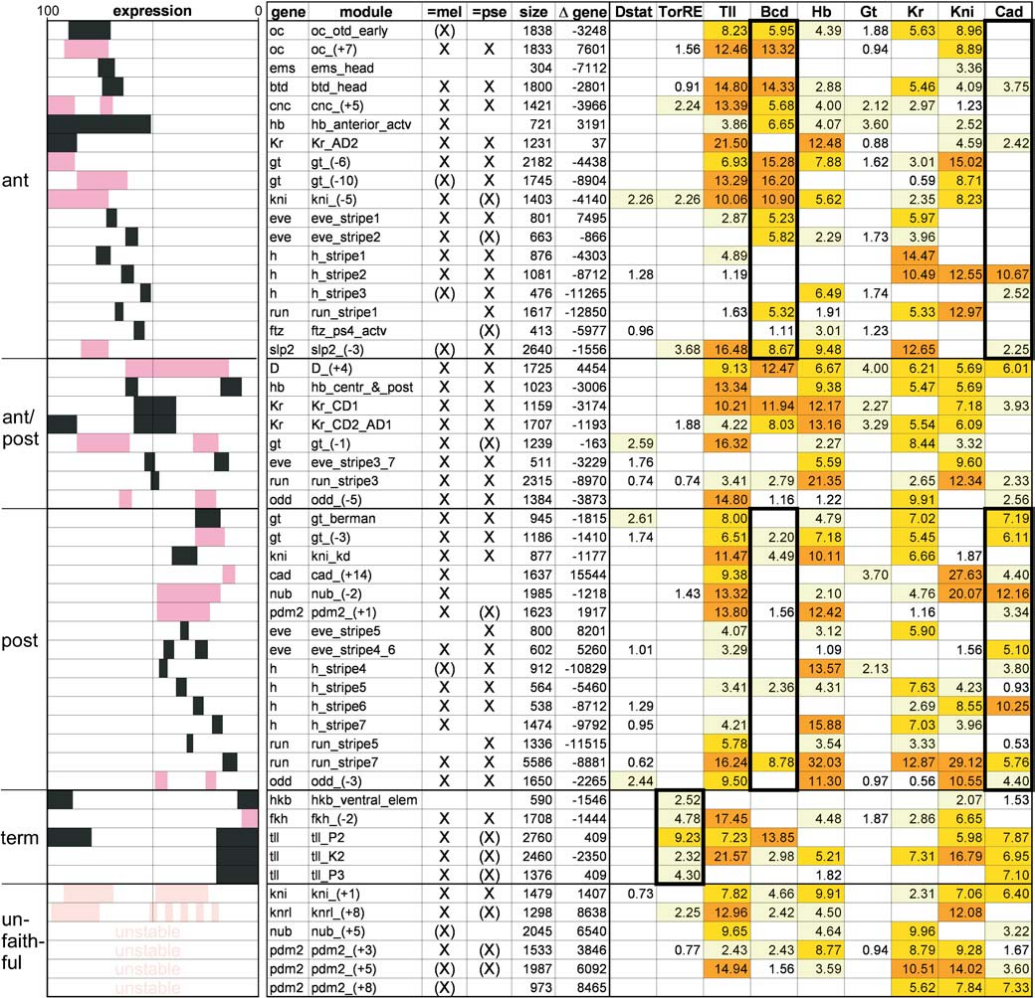
Correlation of Expression Patterns with Module Composition Based on the expression pattern they give rise to, known and newly validated modules are sorted into anterior, posterior, and terminal (if expression bridges the 50% EL line, the module is labeled ant/post), and their binding site composition is evaluated using Ahab output from the mg run. The expression pattern of a module is depicted schematically (anterior = 100% EL, left; posterior = 0% EL, right), followed by name of gene, name of module, recovery as significant prediction (marked by X) or as subthreshold peak (marked by (X)) in D. melanogaster and *D. pseudoobscura,* distance to the gene's transcription start site (negative values denote upstream location), and binding site composition. For references see Dataset S1. Expression patterns of previously known modules are in black, those of newly validated modules are in dark pink, and modules with unfaithful/unstable patterns are in light pink. Binding site composition is given in the form of integrated profile values for individual input factors (see Materials and Methods); higher color intensity emphasizes higher values. Diagnostic features are emphasized by black trim: In anterior modules Bcd sites are overrepresented and Cad sites are underrepresented, while in posterior modules Cad sites are overrepresented and Bcd sites underrepresented. Terminal modules are enriched in TorRE sites.

Under these conditions, Ahab predicts 52 modules within the genomic region of the 29 genes of interest, an average of about two modules per gene. This hit rate represents a 5-fold enrichment compared to the genome-wide rate. Of the 52 predicted modules, 43 are located in intergenic regions, nine in introns, and none in coding regions, indicating a bias of the predictions toward transcriptional control regions. Of the 31 known modules, we recover 22 as significant predictions (score >15; because of overlaps, 20 Ahab predictions cover the 22 known modules), and three overlap with free energy peaks just below the cutoff (Figure 1; cf. Figure 4). In the six cases where Ahab misses known modules completely, the reasons are most likely missing input factors (e.g., *hkb_ventral_element* module; Hader et al. 2000) or a low number of binding sites (e.g., *ems_head* module; Hartmann et al. 2001). The likelihood of recovering 22 modules at random is negligible (*p* < 10^−8^). We also predict 32 novel modules, and we expect at least some predictions with scores below 15 to be functional as well.

**Figure 2:**
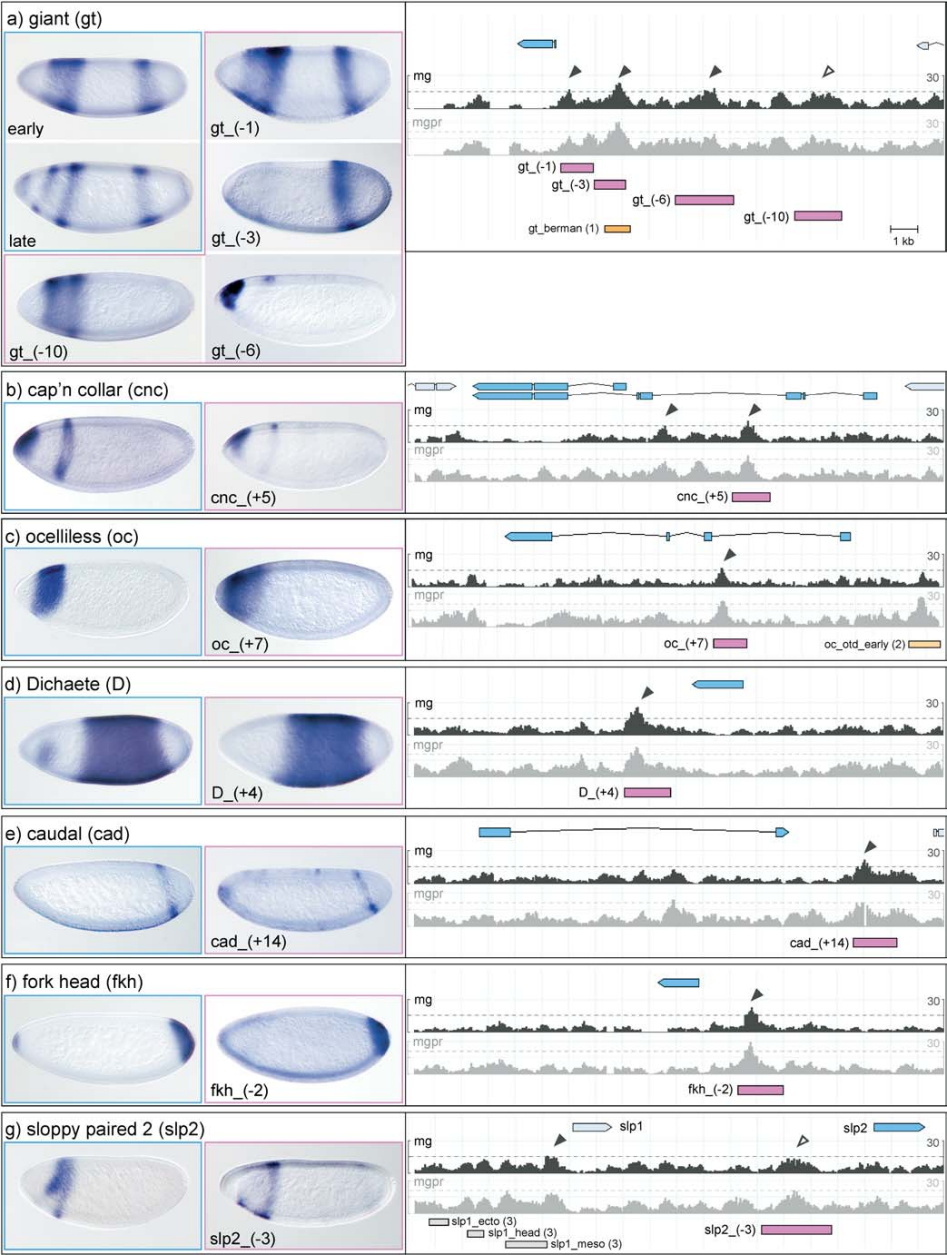
Expression Patterns Driven by Ahab-Predicted Modules I Ahab-predicted modules in the control region of gap and pair-rule genes were tested by fusing putative modules to a basal promoter driving *lacZ* (module-basal promoter-lacZ; Thummel and Pirrotta 1991). The genomic regions, with free energy profiles, for two Ahab runs (mg and mgpr) are shown on the right. The free energy cutoffs are marked by dotted lines; mg run predictions with scores greater than 15 are marked by black arrowheads, tested subthreshold peaks with scores below 15 by open arrowheads. The transcribed region of the locus is marked in blue, the experimentally tested genomic regions are marked by pink bars and named according to distance from transcription start site to middle of the enhancer, and previously known modules are marked by orange bars. The endogenous gene expression is shown on the left (blue frame), the expression pattern driven by the module(s) in the center (pink frame). Embryos are oriented anterior to left, dorsal up. In a few cases, the patterns driven by Ahab-predicted modules are unfaithful to the endogenous gene expression; we distinguish “unfaithful” and insertion-dependent “unstable” patterns. For further description see text. (A) *gt,* (B) *cnc,* (C) *oc,* (D) *D,* (E) *cad,* (F) *fkh,* and (G) *slp2.* References: (1) Berman et al. (2002), (2) Gao and Finkelstein (1998), (3) Lee and Frasch (2000), and (4) Pankratz et al. (1992) and Rivera-Pomar et al. (1995).

**Figure 3:**
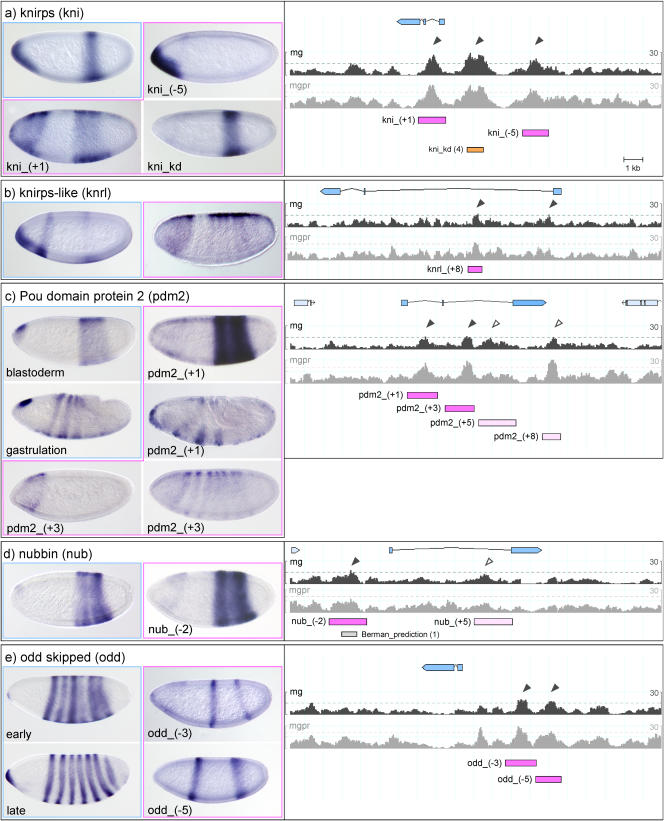
Expression Patterns Driven by Ahab-Predicted Modules II See legend for Figure 2. (A) *kni,* (B) *knrl,* (C) *pdm2,* (D) *nub,* and (E) *odd.*

For experimental validation, we selected 16 module predictions with scores greater than 15 and five with scores below 15 (Figures 2 and 3), located near genes with gap and pair-rule patterns whose control regions had not or only partially been dissected: *cad*, *cap ‘n' collar (cnc), Dichaete (D), fork head (fkh), gt, kni, knirps-like (knrl), nubbin (nub), ocelliless (oc), POU domain protein 2 (pdm2), odd skipped (odd),* and *sloppy paired 2 (slp2)*. We used the free energy profiles to delineate the module and then tested its ability to drive blastoderm expression using a *lacZ* reporter construct (see Materials and Methods). All of the predicted modules we tested drive expression in the blastoderm. However, the faithfulness of the patterns produced by the modules varies. Of the 16 modules with scores greater than 15, 13 produce faithful patterns that reproduce one or more aspects of the endogenous pattern, two produce unfaithful patterns, and one has an unstable, insertion-dependent pattern. Of the five modules with scores below 15, two produce faithful and three produce unstable blastoderm patterns. This indicates that Ahab has excellent success in predicting modules driving blastoderm expression and that the free energy cutoff is well chosen, with few false positives and negatives. The fact that unfaithful or unstable patterns are produced by some of the modules is likely a reflection of the fact that Ahab makes predictions simply on the basis of the total free energy without any explicit rules as to the number and type of factors that have to contribute to the binding. By comparing the composition of modules of different degrees of faithfulness or stability, one can attempt to formulate such rules (see below).

### Using Ahab for the Dissection of Segmentation Gene Control Regions

The gap gene *gt* is initially expressed in two domains in the blastoderm, one anterior and one posterior; as cellularization progresses, the anterior domain splits into two stripes, and, finally, a third expression domain develops at the anterior terminus. We predict three modules*, gt_(−1), (−3),* and *(−6),* all of which we tested; in addition, we tested one subthreshold peak further upstream, *gt_(−10)* (see Figure 2A). We can account for all *gt* pattern elements: the subthreshold *gt_(−10)* faithfully produces the anterior expression, *gt_(−6)* produces the anterior tip expression, and the *gt_(−3)* module produces the posterior expression (cf. Berman et al. 2002). Interestingly, *gt_(−1)* is bifunctional and produces both the anterior and the posterior expression domain.

The gap gene *kni* is expressed in two domains in the blastoderm, one at the anterior tip and one in the posterior, but only the module driving the posterior expression had previously been identified (Pankratz et al. 1992). In addition to the known module *kni_kd,* we predict two additional modules, one further upstream, *kni_(−5),* and one in the first intron, *kni_(+1)*. The *kni_(−5)* module faithfully produces the expression at the anterior tip, while the *kni_(+1)* module drives an imprecise *kni* pattern with an aberrant anterior and an abnormally widened posterior expression domain (see Figure 3A). The sister gene *knrl* is expressed in the same pattern as *kni*. We find two significant predictions in the control region; we tested one, *knrl_(+8),* which produces an unfaithful pair-rule-like pattern (see Figure 3B).

The less well known gap genes *nub* and *pdm2* are both expressed in a broad posterior domain; *pdm2*, but not *nub,* develops a segmental pattern during gastrulation. The control regions of the two genes have not been dissected (Kambadur et al. 1998). We find one significant prediction for *nub, nub_(−2),* and two for *pdm2, pdm2_(+1)* and*(+3). nub_(−2*) faithfully reproduces the posterior expression of the gene (see Figure 3D). For *pdm2, pdm2_(+1)* faithfully reproduces the posterior domain as well as the segmental expression of the gene, while *pdm2_(+3)* produces line-dependent variable patterns of blastoderm expression (see Figure 3C).

The *cad* gene is expressed both maternally and zygotically. Its zygotic expression in the blastoderm consists of a single posterior stripe. We make a single significant prediction, *cad_(+14),* which faithfully reproduces the pattern (see Figure 2E). *fkh* is initially expressed in a single domain at the posterior end, to which a second domain at the anterior end is added later in the blastoderm. We make a single significant prediction, *fkh_(−2),* which faithfully produces the early domain at the posterior end (see Figure 2F). The head gap gene *cnc* is expressed in two domains, an anterior cap and a collar. Our single significant prediction, *cnc_(+5),* faithfully produces the pattern (see Figure 2B). Similarly, the single significant prediction for *oc, oc_(+7),* faithfully produces the single head gap domain of the endogenous gene (see Figure 2C). *D* is initially expressed in a broad domain encompassing the entire segmented portion of the blastoderm embryo, and an anterior patch is added at the end of the blastoderm. The control region of *D* has not been dissected (Sanchez-Soriano and Russell 2000). Our single significant prediction, *D_(+4),* faithfully produces the early blastoderm pattern (see Figure 2D).

Finally, the pair-rule genes: *slp1* and *slp2* are first expressed in a gap-like pattern in the head, followed by expression in seven and then fourteen stripes. The dissection of the upstream region of *slp1* had identified the stripe element but not the gap-like expression in the head (Lee and Frasch 2000). We find a subthreshold peak upstream of *slp2* that nicely reproduces the missing head gap pattern (see Figure 2G). *odd* is first expressed in a pair-rule and then in a segmental pattern and has traditionally been placed among the secondary pair-rule genes, which are thought to generate their pattern through pair-rule input rather than direct maternal/gap input. Surprisingly, we find two significant predictions in the upstream region of the gene, *odd_(−3)* and*(−5).* Both these modules drive expression in two stripes: *odd_(−3)* drives expression in stripes 3 and 6, while *odd_(−5)* drives expression in stripe 1 and a broader region encompassing stripes 5 and 6 of the endogenous pattern (see Figure 3E). This behavior is reminiscent of the two-stripe modules of *eve (eve_stripe3_7* and *eve_ stripe4_6).* Thus, at least four of the seven *odd* stripes are formed as individual stripes by maternal/gap input rather than as a complete seven-stripe pattern, indicating that *odd* has primary pair-rule character.

Overall, our experimental validation demonstrates that Ahab is highly successful in predicting modules that drive patterned expression in the blastoderm. The algorithm finds missing modules that complement existing ones to collectively produce the expression pattern of a gene and identifies, with surprising accuracy, relevant modules in previously undissected control regions. Most of the modules faithfully produce pattern elements of the endogenous gene, suggesting that our delineation of modules, which is based on the free energy profile of the prediction, is generally quite accurate.

### Module Composition and Pattern of Expression

Ahab's success in finding modules encouraged us to examine in greater detail its prediction of the binding site content of modules. We sought to examine whether the expression patterns of the previously known and our newly tested modules correlate with their composition.

In its optimization procedure, Ahab fits all input factors simultaneously to the genomic region of interest, while experimental sites for transcription factors are typically determined in the absence of any competition. Ahab reports binding site composition in the form of integrated profile values, which tally the fractional occupancy of sites for a given factor, and are thus a measure of the strength of binding by this factor (see Materials and Methods). In order to gauge the accuracy of Ahab predictions of module composition, we examined how well Ahab performs in recovering known binding sites (for detailed description see Materials and Methods). Overall, the recovery of known sites ranges from 50% to 100%, with the most specific factors/position weight matrices showing the best recovery. The missed sites are typically weak and are not misattributed to other factors but rather to background. Thus, Ahab should provide a reliable profile of module composition.

In order to correlate the binding site composition with the ap expression pattern of the modules, we charted the previously known modules and all the newly validated modules with faithful expression and sorted them according to their expression along the ap axis (see Figure 4). We ask which, if any, features are diagnostic.

### The Maternal Factors

In anterior modules (driving expression at 50%–100% egg length ), Bcd sites are overrepresented and Cad sites underrepresented (see Figure 4), including seven known and six newly tested modules. In posterior modules (driving expression at 0%–50% EL), Bcd sites are underrepresented and Cad sites overrepresented, including five known and five newly tested modules. Finally, in terminal modules (driving expression at 0%–20% and 80%–100% EL), TorRE sites are strongly overrepresented, including four known and one newly tested modules. In addition to the TorRE-terminal signature, terminal modules expressed at the anterior terminus often contain Bcd sites, and those expressed at the posterior end, Cad sites. Thus, there is a strong positive correlation between the expression pattern of the module and the maternal input they receive, supporting the general interpretation that the maternal factors act as transcriptional activators in their realm of expression.

**Figure 5:**
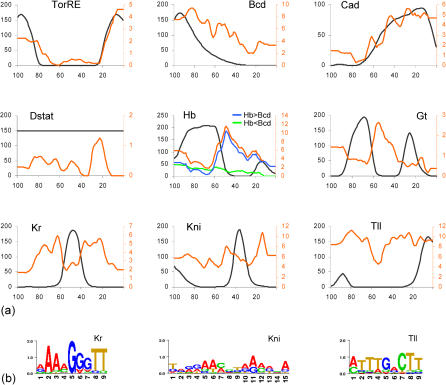
Ap Distribution of Binding Sites and Cognate Input Factors (A) Plots depict distribution of input factors (black) along the ap axis (anterior tip = 100, posterior tip = 0) (based on Myasnikova et al. [2001]) and the average number of binding sites (as measured by integrated profile values; Figure 4) found in all modules driving expression at a given percent EL (red) (see Materials and Methods). For TorRE, Bcd, and Cad, the distributions of binding sites and input factors are positively correlated. For Hb, Gt, and Kr, the distributions are negatively correlated; note that the number of binding sites is particularly high in modules expressed adjacent to the expression domain of these factors. In the case of Hb, modules with more Hb sites than Bcd sites (blue) show negative correlation with input factor distribution, and modules with fewer Hb sites than Bcd sites (green) show positive correlation, indicating bimodal function of Hb. For Kni and Tll, no clear correlations are found, possibly because of the unspecificity of their weight matrices. (B) Information scores of the Kr, Kni, and Tll weight matrices.

To take a closer look at this relationship, we computed for each input factor and for every position along the ap axis the average number of binding sites found in the modules driving expression at that position. We plotted this number as a function of ap position and compared the resulting curve with the input factor distribution as determined by Reinitz and coworkers (Myasnikova et al. 2001) (Figure 5A). For TorRE, the distribution of binding sites beautifully follows the expression profile of the input factor (as inferred from expression of its negative regulator, Capicua), indicating that binding sites are present almost exclusively where the cognate factor is active. The distributions of Bcd and Cad binding sites broadly conform with the anterior and posterior gradients of their respective input factors. The rise in the curves at the posterior terminus for Bcd and at the anterior terminus for Cad is caused by terminal modules expressed at both ends of the embryo. Overall, for the maternal activators, the binding site composition of modules is well fitted to the input factor distribution.

### The Gap Factors

The situation regarding the gap factors is more complex. When examining the distributions of Hb, Gt, and Kr binding sites and comparing them with the input factor distributions, we clearly find an anticorrelative relationship: The number of sites is lower in regions where the cognate factor is present, and higher in regions where the factor is absent (Figure 5A). Remarkably, the number of sites is particularly high in regions immediately adjacent to the expression domain of the factor. These findings are consistent with the experimental evidence that gap factors act as repressors. Thus, modules which have many sites efficiently suppress expression within the domain of the input factor, and permit expression only outside the domain. The great majority of modules conform to this anticorrelative relationship; we can therefore conclude that, overall, repression is the prevalent mode of action for these gap factors.

However, we do find some modules that appear to be coextensively expressed with the presumptive repressors. One possible explanation is that the input factor has a different mode of action in these modules, that is, instead of repression it may mediate activation. Hb appears to be an example for such a switch in the mode of action. We find many modules with a small number of Hb sites that are coextensively expressed with Hb in the anterior, and it has been shown experimentally that Hb function is context dependent: Repressor function has been demonstrated for several posterior modules (e.g., *kni_kd, eve_stripe3_7,* and *eve_stripe4_6*) (Pankratz et al. 1992; Fujioka et al. 1999), while activator function has been demonstrated for several anterior modules (e.g., *hb_anterior, Kr_CD1,* and *eve_stripe2*) (Treisman and Desplan 1989; Hoch et al. 1990; Small et al. 1991; Stanojevic et al. 1991). It is thought that Hb is converted from a repressor to an activator by the concurrent presence of homeobox factors such as Bcd (Zuo et al. 1991; Simpson-Brose et al. 1994). We examined the composition of these two sets of known modules and found that in the posterior modules, in which Hb acts as a repressor, the profile values of Hb exceed those of Bcd, while in anterior modules, in which Hb acts as an activator, the profile values of Hb are lower than those of Bcd. When we apply the simple rule suggested by this observation to all modules containing Hb sites, we find that it significantly improves the picture: the Hb>Bcd (Hb as repressor) set is strongly negatively correlated with Hb factor expression, while the Hb<Bcd (Hb as activator) set is positively correlated with Hb factor expression (the only exception is the *D_(+4)* module, which drives expression in a broad domain straddling the 50% EL line). Thus, the global distribution profile of Hb sites can largely be explained by introducing a simple contextual rule.

By contrast, for Gt and Kr, the number of modules expressed coextensively with the input factor is comparatively small. In the case of Gt, all experimental evidence points to its acting as a repressor. Increasing the spatiotemporal resolution of the plot to reflect the modulation of Gt expression over time may be sufficient to account for the presence of Gt sites in at least some of the potentially “noncompliant” modules (*cnc_(+5), oc_(+7), oc_otd_early,* and *hb_ant*). In the case of Kr, context-dependent function has been suggested, but mostly based on tissue culture experiments (Sauer et al. 1995; La Rosee et al. 1997; La Rosee-Borggreve et al. 1999). The four potentially noncompliant modules (*Kr_CD2, run_stripe3, nub_(+5), D_(+4))* are clearly expressed coextensively or overlapping with the Kr input factor. Since the average number of binding sites is low in these modules, it is possible that Kr acts as a repressor but that this manifests itself only in a reduced expression level. In fact, the *Kr_CD2* module has been noted to be more weakly expressed than its sister module *Kr_CD1,* which lacks Kr sites (Hoch et al. 1990), but there are too many other differences in their binding site composition to draw any firm conclusions. These noncompliant modules provide a solid experimental platform for resolving the issue of whether or not Kr truly switches its mode of action in vivo.

Finally, for Kni and Tll, most experimental evidence points to repression, but context-dependent activation has been suggested in a few cases (Langeland et al. 1994; Margolis et al. 1995; Kuhnlein et al. 1997; Hartmann et al. 2001). As noted at the beginning, the weight matrices for both factors are fairly unspecific (Figure 5B), resulting in a lower level of confidence in the predictions, which typically show a large number of binding sites. When plotting binding site and input factor distributions, no clear positive or negative correlations are visible (Figure 5A), suggesting either strong context-dependent function—which is not really supported by the extant literature—true indiscriminate binding, or simply poor binding site information.

### Unfaithful Modules

In our experimental tests, we found a few novel modules that drive unfaithful patterns. Can we understand their behavior based on the composition profile of the module? We observed two flavors of unfaithful expression: strong invariant and weak variable. The *kni_(+1)* module is an example of the former: It drives expression in a posterior domain that is wider than the endogenous pattern (see Figure 3A). When compared to the faithful *kni_kd* module, *kni_(+1)* contains the same types of binding sites, but with different profile values: The profile values for the activator Cad are higher and the ones for the repressors Hb, Kr, and Tll are lower (see Figure 4). This suggests that an increase in activator binding together with a decrease in binding by adjacently expressed repressors may be responsible for the widening of the posterior domain. The *pdm2_(+3)* module is an example of weak and unstable expression (see Figure 3C), which we find more often when analyzing subthreshold peaks. Such modules typically suffer from a reduced number of activator sites and an increase in sites for coextensively expressed repressors (see Figure 4). Thus, the two flavors of unfaithful patterns, strong invariant and weak variable, appear to correlate with the ratio of activator to repressor sites in the module and the degree to which the distributions of the relevant input factors are compatible. Further experimental and computational work will be required to determine precise module composition rules, but both faithful and unfaithful modules can contribute to defining them.

### Evolutionary Conservation

The availability of the Drosophila pseudoobscura genome makes it possible to ask how well segmentation modules are conserved. In a previous study, Emberly et al. (2003) showed that the degree of sequence conservation between D. melanogaster and D. pseudoobscura is not significantly higher in known segmentation modules than in surrounding noncoding regions, suggesting that sequence conservation per se is not sufficient to identify such modules. We obtain the same result for our Ahab predictions (data not shown). However, when we run Ahab over the aligned segmentation gene control regions in *D. pseudoobscura,* using D. melanogaster weight matrices as input, we recover as significant predictions about the same number of known modules as in *D. melanogaster,* indicating that there is substantial *functional* conservation (see Materials and Methods). However, only 24 of the 35 known and newly validated modules that are recovered in D. melanogaster also score as significant predictions in *D. pseudoobscura,* with an additional seven as subthreshold peaks (see Figure 4). Conversely, four subthreshold D. melanogaster modules are recovered as significant predictions in *D. pseudoobscura,* and three known modules are recovered only in D. pseudoobscura. Thus, modules with maternal/gap input appear to be in some evolutionary flux, which needs to be taken into consideration if evolutionary conservation is employed as a tool in module discovery.

### Regulatory Input within the Segmentation Gene Hierarchy

Given Ahab's success in predicting modules with maternal and gap input, we decided to expand the analysis to the entire segmentation gene network and explore the algorithm's performance when less well defined binding site information is available. To this end, we included the control regions of a total of 48 genes: To the genes with gap-like and pair-rule patterns, we added segment-polarity and homeotic genes (for references see Dataset S1). Concurrently, we expanded the set of binding site inputs. The maternal and gap factors were used as before (mg run). In addition, we collected binding site information from the literature for the pair-rule factors Hairy (H), Even skipped (Eve), Runt (Run), Fushi tarazu (Ftz), Ftz transcription factor 1 (Ftz-f1), Paired (Prd), and Tramtrack (Ttk) (Dataset S2). For all these factors the available binding site information is generally less extensive and relies less on in vivo and more on in vitro experiments such as Selex. This again has the consequence that the weight matrices are artificially more specific, resulting in the prediction of fewer sites but higher scores for a match. The pair-rule factors were run by themselves (pr run) and in combination with the maternal and gap factors (mgpr run), with window size 500 and background model 2.

In the combined control regions of the entire set of 48 segmentation genes, which total 1.7 Mb in length, we find 82 significant peaks for the mg run (score >15), 56 for the pr run (score >15), and 69 for the mgpr run (score >22, cutoff set to equal genome-wide mean plus four standard deviations), in total 145 distinct putative modules, an average of approximately three per gene. Interestingly, the mg run and pr run peaks are completely nonoverlapping. We determined the relative contribution of maternal/gap and pair-rule input to each predicted module by evaluating its binding site composition as revealed by the mgpr run, i.e., using all input factors. Modules were classified into four types: maternal/gap driven, pair-rule driven, or driven by both but with bias towards maternal/gap input or pair rule input (see Materials and Methods).

The number and types of modules found within the control region of each target gene are shown in Figure 6B. For the genes with gap-like expression, maternal and gap input strongly predominates; for pair-rule and segment-polarity genes, pair-rule input predominates. The homeotic genes receive both types of input. This global result reflects very well the overall regulatory structure of the segmentation gene network. However, we find interesting exceptions to the global rules. Among the pair-rule genes, *odd* stands out as receiving unexpectedly strong maternal/gap input. *odd* is expressed in a pair-rule and then segment-polarity pattern (Coulter et al. 1990) and has traditionally been placed among the secondary pair-rule genes (Klingler and Gergen 1993; Pankratz and Jäckle 1993; Pick 1998). But as our dissection reveals (see Figure 3E), *odd* receives strong maternal/gap input and generates at least four of its seven stripes via two-stripe modules, suggesting that it in fact belongs to the primary pair-rule tier. In addition, as noted above, the control region of the secondary pair-rule gene *slp2* contains a subthreshold peak with maternal/gap input that drives its early gap-like expression in the head region (see Figure 2G).

**Figure 7:**
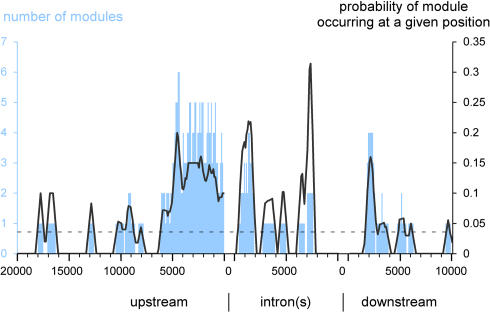
Genomic Position of Modules Position of modules predicted by the Ahab mg run relative to the transcription start site of the cognate loci; predictions for the homeotic genes are excluded. The number of modules found at a given position is shown in blue. The black line indicates the probability of a module occurring at a given position (calculated by dividing the number of modules at a given position by the number of control regions extending to that position). The stippled black line shows that probability if modules were randomly distributed. Modules with maternal/gap input are clustered within the first 6 kb upstream, in the first 2 kb of intronic space, and around 2 kb downstream (measured from the end of the gene).

Finally, we also examined the position of known and predicted modules relative to the transcription start site of the gene (Figure 7) We found that maternal/gap-driven (mg run) modules are strongly biased toward the proximal upstream region (−6 to 0 kb), the first 2 kb of intronic space, and the proximal downstream region (+2 to +4 kb). This clustering is found for the gap, pair-rule, and segment-polarity genes, whose genomic organization is typically simple, but not for the homeotic genes, which typically have much larger control regions and multiple large introns, with wide scattering of predicted modules. For pair-rule-driven (pr run) modules, a similar though less pronounced clustering is observed (data not shown).

## Discussion

In this study we have demonstrated that the Ahab algorithm can be used successfully for two purposes: the prediction of novel segmentation modules within genomic sequence and the prediction of module binding site composition. The computational analysis of control regions with Ahab dramatically improves the efficiency of the experimental dissection, allowing us to significantly increase the number of validated *cis-*regulatory elements from 31 to 46 and to provide effective de novo dissections for ten segmentation genes. Two principal factors contribute to this success. First, the existing experimental data for the segmentation gene network provide a rich substrate for the computational effort. Second, the biochemistry underlying the regulation of transcription, that is, the binding of transcription factors to DNA, is well described by equilibrium thermodynamics (von Hippel and Berg 1986; Berg and von Hippel 1987, 1988a, 1988b; Ptashne and Gann 2001), and thus Ahab's use of equilibrium conditions to predict the number, type, and occupancy of binding sites within a window of genomic sequence mimics the intrinsic process.

The global analysis of the segmentation gene hierarchy shows that the prevalence of maternal/gap input strongly correlates with gap-like expression, while the prevalence of pair-rule input strongly correlates with segmental expression. Integrating the inputs over all modules within the control region of a gene provides a reliable indication of its type of expression pattern and position within the hierarchy. In fact, the integrated predictions are so accurate as to pinpoint abnormalities in the gene classification, such as the known head gap function of *slp2,* and also the hitherto unknown primary pair-rule character of *odd*. Since our knowledge of input factor sites is incomplete (particularly regarding the pair-rule factors), these positive results are likely to reflect the redundant and combinatorial nature of the input.

Ahab performs well not only in identifying modules, but also in predicting their composition, thus permitting an analysis of binding site composition under uniform criteria for the entire set of known and newly validated maternal/gap-driven modules. Gene expression studies in mutant embryos have revealed the global regulatory interactions within the segmentation gene network (St Johnston and Nusslein-Volhard 1992; Pankratz and Jäckle 1993; Rivera-Pomar and Jackle 1996; Furriols and Casanova 2003), but are not suited to uncover redundancies within the network or to separate direct from indirect effects. This becomes possible by examining the inputs into the *cis-*regulatory modules. We find that the vast majority of the modules expressed in the early blastoderm contain maternal factor sites, which strongly suggests that the maternal gradient systems of Cad, Hb, Bcd, and Torso (through its transcriptional effectors) have most, if not all, of the early zygotic patterning along the ap axis under their direct control. Together with the strong interdependence of the maternal gradient systems, this massively parallel output would explain the coordinated and long-range effects on segmentation gene expression patterns that are observed when maternal factors are titrated up or down through genetic manipulation.

Further, by correlating the binding site content of modules driving expression at a given position with input factor distributions, we are able to infer the mode of action for six of the nine factors and to show that modules are generally well fitted to the distributions of their positive and negative input factors. The maternal factors act as activators within their domain of expression, while the gap factors act largely as repressors. This overall result confirms previously existing data and demonstrates that the rules gleaned earlier from rather small datasets generalize very well over the entire set. Interestingly, our data also provide support for the idea that Hb functions in a bimodal fashion and suggest a simple rule for its context-dependent switch from repression to activation. Modules with few Hb and many Bcd sites drive expression in the anterior half of the embryo, while modules with more Hb than Bcd sites do not. Depending on module composition and Bcd availability, Hb can thus activate transcription; this Bcd/Hb synergy could serve to bolster transcriptional activation in regions where Bcd levels taper off. For Kni and Tll, the mode of action cannot be assessed on the basis of the extant binding site information.

The comparison of modules with faithful and unfaithful or unstable patterns provides some interesting additional clues for composition rules, such as the ratio and compatibility of activator and repressor sites. However, to address the question of how the precise domain boundaries are established within a given region of the embryo, a more detailed examination of composition rules and of the internal organization of modules will be needed, specifically of rules governing the number, affinity, spacing, and arrangement of binding sites. This analysis will require different types of experimentation as well as additional computational analysis.

The performance of Ahab is influenced by a number of parameters, but the most important is the quality of the input factor weight matrices. To further improve weight matrices, more sites for undersampled factors will have to be collected (D-Stat and Gt), and existing sites for the unspecific factors will have to be scrutinized (Kni and Tll). More importantly, the relative affinity of binding sites for their factor will have to be measured in a more comprehensive fashion. The ideal experiment would measure, under identical conditions, the relative binding affinity of the consensus sequence to all possible single base mutations in the consensus binding site (Benos et al. 2002). For some segmentation modules, the currently predicted binding site composition is clearly insufficient to explain their expression, indicating that some of the relevant input has not been characterized. We have experimented with motif-finding algorithms and found that novel, biologically functional binding motifs can be identified by searching for locally overrepresented motifs within known modules and filtering out the known input factor binding sites (see Rajewsky et al. 2002; J. F., M. P., M. D. S., and U. G., unpublished data), which suggests that computational methods can also assist the identification of novel input factors.

With an Ahab run that recovers 70% of the known modules with predominant maternal/gap input, we predict another 32 putative modules in the control regions of gap and pair-rule genes. Most of these look plausible in terms of genomic location and composition, and as our validation shows, many drive blastoderm expression that faithfully reproduces the endogenous pattern of the gene. However, we also found modules whose expression does not match the endogenous pattern (unfaithful/unstable) or whose composition does not suggest any coherent expression pattern (e.g., no activator sites); among the latter are some predictions dominated by Kni and Tll sites, which are potentially problematic because of the unspecificity of their weight matrices. The apparently anomalous modules could drive expression at later stages of development or could simply be artifacts of improper delineation or missing relevant input. A more intriguing possibility is that some of these modules are in evolutionary transit—nascent or dying. Such modules might be held in check by relatively few point mutations (“pseudo” modules), by nearby insulator elements, or by restricted access to the basal promoter when competing with the functional modules. The effort to discover the true nature and function of these anomalous modules will be aided by the computational and experimental comparison of corresponding modules in D. melanogaster and D. pseudoobscura.

## Materials and Methods

### 

#### Position weight matrices and Ahab runs

When possible, previously compiled position weight matrices were used: for Bcd, Hb, Cad, TorRE, Kr, Kni, and Tll (Rajewsky et al. 2002), and for Ftz, Prd_HD, and Ttk (Papatsenko et al. 2002). For H, Run/CBF, and D-Stat, we directly used in vitro selection data (Melnikova et al. 1993; Van Doren et al. 1994; Yan et al. 1996). For Eve_HD, the alignment was taken from the literature (Hoey et al. 1988), for Gt, Eve_t2, and Ftz-f1, footprinted sites from the literature were aligned (Hoey et al. 1988; Biggin and Tjian 1989; Ueda et al. 1990; Jiang et al. 1991; Capovilla et al. 1992; Fujioka et al. 1996; Florence et al. 1997; Yu et al. 1997; Shimell et al. 2000). The binding sites, alignments, and weight matrices used plus references are listed in Dataset S2. For description and mathematical details of the algorithm, see Rajewsky et al. (2002). All runs were carried out on *Drosophila* genome sequence Release 2 after masking tandem repeats in the genomic sequence as described in Rajewsky et al. (2002). Control regions were defined as the sequence surrounding a gene and limited by the two flanking genes, up to a maximum of 20 kb upstream and 10 kb downstream, and with a buffer for the flanking genes of 2 kb upstream and 1 kb downstream. For the homeotic genes, no maximum for the upstream or downstream extension of the control region was imposed.

#### Mapping of known modules

The genomic position of known modules was derived from literature (Papatsenko et al. 2002; Rajewsky et al. 2002) or mapped to genomic sequence from the literature using restriction sites, PCR primers, or distances relative to transcription start site. For a complete list and description see Dataset S3.

#### Significance of Ahab predictions

To assess the significance of Ahab module predictions, we calculated the overlap between predictions and known modules in basepairs, and compared it with the overlap achieved when predictions are randomly placed within the delineated control regions (minus masked and coding sequence). We failed to match the actual overlap through 10^8^ randomizations, resulting in an estimate of *p* < 10^−8^ for the significance of the recovery of known modules by Ahab. When we remove from the calculation the 13 modules that were used for the construction of weight matrices *(Kr_CD1, Kr_CD2_AD1, eve_stripe3_7, eve_stripe2, h_stripe5, h_stripe6, hb_anterior_actv, hb_central_&_posterior_actv, kni_kd, oc_head, tll_K2, tll_P2,* and *tll_P3*), along with the *kni, hb,* and *tll* control regions, which contain no additional annotated modules, we find *p* = 4.9 × 10^−6^.

#### Ahab recovery of known binding sites

The experimental binding sites that define our weight matrices are derived from a variety of in vitro experiments that typically neglect competition between transcription factors, whereas Ahab, in its prediction of binding sites, fits all factors simultaneously. To gauge whether Ahab can be used as a predictor of module composition, we examined what fraction of known binding sites the algorithm recovers. The only free parameter in the comparison is the profile value (between 0 and 1), which measures the fractional occupancy of a site by its factor; a profile value of 1 means that a site is always occupied by its factor. A site was scored as found if the prediction exceeded a certain profile value cutoff and overlapped the experimental footprint by more than 50%. Table 1 correlates the recovery of sites with the specificity of the weight matrices for two profile value cutoffs. Overall, the recovery ranges from 50% to 100%, with the most specific factors/matrices showing the best recovery.

**Table 1:**
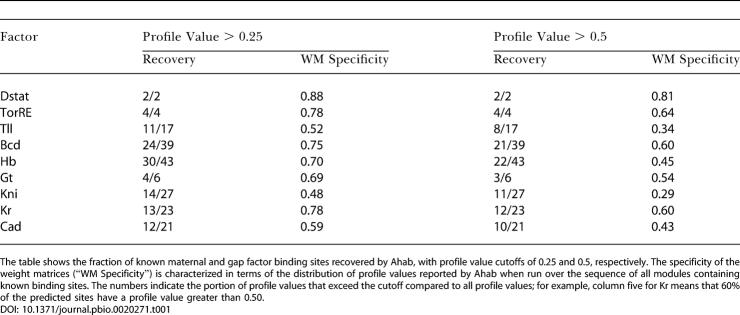
Recovery of Known Binding Sites

The table shows the fraction of known maternal and gap factor binding sites recovered by Ahab, with profile value cutoffs of 0.25 and 0.5, respectively. The specificity of the weight matrices (“WM Specificity”) is characterized in terms of the distribution of profile values reported by Ahab when run over the sequence of all modules containing known binding sites. The numbers indicate the portion of profile values that exceed the cutoff compared to all profile values; for example, column five for Kr means that 60% of the predicted sites have a profile value greater than 0.50

We further examined whether Ahab misses known sites by misclassification. We found that Ahab generally does not misattribute the missing sites to another factor. A cogent example is provided by Cad and Hb, which have very similar binding sites containing an oligoT stretch. Surprisingly, none of the 21 Hb sites that were missed at a profile value cutoff of 0.5 were misclassified as Cad; conversely, only one of the 11 missed Cad sites was classified as Hb. This discrimination is far better than that achieved by a simple weight matrix scan over the same modules: for this scan, we counted information scores greater than five, which is the score of the weakest experimental binding sites, and overlaps between matrix and binding site of 50% or more. The matrix scans correctly classified 29/43 Hb sites and 16/21 Cad sites; but misclassified five Hb sites as Cad and two Cad sites as Hb. Taken together, Ahab finds the majority of known binding sites and rarely misclassifies; it is thus a reliable indicator of module composition. A complete listing of the integrated profile values reported by Ahab for known, newly validated, and predicted modules is available in Dataset S6 (mg run) and Dataset S7 (mgpr run).

#### Recovery of modules in *Drosophila pseudoobscura*

To assess the conservation of known and Ahab-predicted modules, we aligned D. melanogaster and D. pseudoobscura genomic sequence as described in Emberly et al. (2003) and ran Ahab over the aligned D. pseudoobscura control regions, with D. melanogaster weight matrices as input and with cutoffs for significant predictions (15 in D. melanogaster) and subthreshold peaks (12 in D. melanogaster, equal to genome-wide mean plus three standard deviations) set to obtain equivalent numbers of predictions in *D. pseudoobscura. D. pseudoobscura* predictions were then mapped to D. melanogaster coordinates and examined for overlap with the known and predicted D. melanogaster modules.

#### Processing of Ahab output and module classification

To associate predictions from different Ahab runs, each run was processed and the highest point on the free energy plot within an interval of the window size was marked as a “peak.” Peaks are thus spaced by at least the window size. Peaks in two different runs correspond if they are closer than half the window size; their correspondence is unique and order independent. For the three-way comparison, the mg and pr runs were separately compared to the mgpr run. In no case did mg run and pr run peaks correspond without at least one of them matching a mgpr run peak.

For the purposes of broadly classifying predicted modules as to type of input, we defined four classes: mostly maternal/gap input, mostly pair-rule, and mixed input but with a bias towards maternal/gap or pair-rule. Two classification methods were used. The first relied on a single Ahab run with all factors (mgpr run) and then compared the sum of the maternal/gap factor profile values for a given module with the sum of the pair-rule profile values, after normalization to make the mean and standard deviation of maternal/gap profile values over all peaks equal to the mean and standard deviation of all pair-rule profile values. An alternative scheme used the free energy plots for the three runs (mg, pr, and mgpr), identified corresponding peaks, and then compared their rank in the different runs. The two methods yielded very similar results.

Since Ahab does not adapt its window size to the data, modules that are wider than the window size needed to be delineated to be captured accurately. To this end, we defined the start of the module as the first local maximum in the free energy plot that is above the cutoff. The end point was initialized as the other end of the corresponding Ahab window. The plot was then scanned from left to right, and when another local maximum or rise in window score above the cutoff was encountered, the end point of the module was reset as the end of the corresponding window. The sequence of all delineated predicted modules is available in Dataset S5.

#### Molecular biology and RNA in situ hybridization

Module predictions were tested as follows. The module was delineated within the genomic sequence as described above and further expanded to include good primer sites for touch-down PCR. Primers were designed following manufacturer's guidelines (Clontech, Palo Alto, California, United States), restriction sites (Xba, Asp718) were added for subsequent cloning. Genomic PCR products were cloned into TOPO (Invitrogen, Carlsbad, California, United States), sequenced to confirm identity, and subcloned into Casper hs43ßGAL (Thummel and Pirrotta 1991). A Fasta file with the primers and cloned regions is available in Dataset S4.

Transgenic fly strains were generated using standard methods. For each construct three independent insertions were analyzed for expression patterns by RNA in situ hybridization with a *lacZ* probe. RNA in situ hybridizations were carried out as described by Noordermeer and Kopczynski (http://www.fruitfly.org/about/methods/RNAinsitu.html).

#### Delineation of protein and transcript patterns

The protein expression profiles of the maternal and gap input factors were obtained from http://flyex.ams.sunysb.edu (Myasnikova et al. 2001) (temporal class 4, 10% strip, normalized and registered by FRDWT, averaged over 5% EL). In cases where these data were not available, input factor expression profiles were inferred from literature (D-Stat) or our own data (TorRE, measured by expression of the negative regulator Capicua). The output transcript patterns of segmentation gene modules were determined using images of our own RNA in situ hybridizations of blastoderm embryos, and complemented by data from the literature. Embryos were viewed in the sagittal plane, and the intersection of the domain boundaries with the longitudinal axis was determined and calculated as percent EL. Measurements were performed using the Zeiss (Oberkochen, Germany) Axiovision 3.1 measurement tool and averaged over 2–5 embryos. A complete listing of the references for the expression patterns of segmentation genes is found in Dataset S1. To generate the plots in Figure 5A, we calculated, for every input factor and for every position along the ap axis, the average of the integrated profile values reported by Ahab for the modules driving expression at that position. Values were calculated in 1% EL increments, then averaged over 5% EL.

## Supporting Information

The Gbrowse display of free energy profiles for genome-wide Ahab runs (mg, pr, mgpr) can be viewed at http://edsc.rockefeller.edu/cgi-bin/gbrowse_ms/cgi-bin/gbrowse?src=fly.

Dataset S1 Segmentation Genes Referred to in This Study The dataset gives name, symbol, flybase identifier, and references for expression pattern, control region dissection, and binding site information.
(178 KB DOC).

Dataset S2 Compilation of Position Weight Matrices and Binding Sites Used in This Study (8 KB TXT).

Dataset S3 Sequence Information for Known Segmentation Modules in Fasta Format (80 KB TXT).

Dataset S4 Sequence Information for Transgenic Constructs Used in This Study in Fasta Format (37 KB TXT).

Dataset S5 Sequence Information for Ahab-Predicted Modules in the Control Regions of 48 Segmentation Genes Data based on mg run, Fasta format.
(53 KB TXT).

Dataset S6 Profile Value Output for Ahab Mg Run Input: Bcd, Hb, Kr, Gt, Kni, Tll, Cad, TorRE, and Dstat. Performed over defined sequences of known modules (Dataset S3), tested constructs (Dataset S4), and Ahab-predicted modules (Dataset S5).
(62 KB TXT).

Dataset S7 Profile Value Output for Ahab Mgpr Run Input: Bcd, Hb, Kr, Gt, Kni, Tll, Cad, TorRE, Dstat, H, Eve_HD, Eve_t2, Run, Ftz, Ftz-f1, Ttk, and Prd_HD. Performed over defined sequences of known modules (Dataset S3), tested constructs (Dataset S4), and Ahab-predicted modules (Dataset S5).
(77 KB TXT).

### Accession Numbers

The FlyBase (http://flybase.bio.indiana.edu) accession numbers for the genes and gene products discussed in this paper are Bcd (FBgn0000166), Cad (FBgn0000251), Capicua (FBgn0028386), *cnc* (FBgn0000338)*, D* (FBgn0000411), D-Stat (FBgn0016917), Eve (FBgn0000606), *fkh* (FBgn0000659)*,* Ftz (FBgn0001077), Ftz-f1 (FBgn0001078), Gt (FBgn0001150), H (FBgn0001168), Hb (FBgn0001180), Kni (FBgn0001320), *knrl* (FBgn0001323)*,* Kr (FBgn0001325), *nub* (FBgn0002970), *oc* (FBgn0004102), *odd* (FBgn0002985)*, pdm2* (FBgn0004394), Prd (FBgn0003145), Run (FBgn0003300), *slp2* (FBgn0004567), Tll (FBgn0003720), TorRE (cf. FBgn0003733), and Ttk (FBgn0003870).

## Abbreviations

ap: anterior-posterior
Bcd: Bicoid
Cad: Caudal
*cnc*: *cap 'n' collar*
*D*: *Dichaete*
D-Stat: Stat92E
EL: egg length
Eve: Even skipped
*fkh*: *fork head*
Ftz: Fushi tarazu
Ftz-f1: Fushi tarazu transcription factor 1
Gt: Giant
H: Hairy
Hb: Hunchback
Kni: Knirps
*knrl*: *knirps-like*
Kr: Kruppel
mg run: maternal/gap run
mgpr run: combined maternal/gap and pair-rule run
*nub*: *nubbin*
*oc*: *ocelliless*
*odd*: *odd skipped*
*pdm2*: *POU domain protein 2*
pr run: pair-rule run
Prd: Paired
Run: Runt
*slp2*: *sloppy paired 2*
Tll: Tailless
TorRE: Torso-response element
Ttk: Tramtrack
