# Supplementary material for: Transcriptional Control in the Segmentation Gene Network of Drosophila
Source: PLoS Biol. 2004 Aug 31;2(9):e271. doi: 10.1371/journal.pbio.0020271 (PMC514885; doi:10.1371/journal.pbio.0020271)
Supplement: Dataset S1 — The dataset gives name, symbol, flybase identifier, and references for expression pattern, control region dissection, and binding site information. (178 KB DOC). [file pbio.0020271.sd001.doc]

| **Gene name** | **Symbol** | **Flybase ID** | **Pattern reference** | **Control region reference** | **Input reference** |
| --- | --- | --- | --- | --- | --- |
| abdominal-A | abd-A | FBgn0000014 | Karch et al. 1990 | Shimell et al. 2000 |  |
| Abdominal-B | Abd-B | FBgn0000015 | Celniker et al. 1989 |  |  |
| Antennapedia | Antp | FBgn0000095 | Levine et al. 1983 |  |  |
| bicoid | bcd | FBgn0000166 | Driever and Nusslein-Volhard 1988; Driever and Nusslein-Volhard 1988 | ND | Rajewsky et al. 2002 |
| buttonhead | btd | FBgn0000233 | Wimmer et al. 1993 | Wimmer et al. 1995 |  |
| cap ‘n’ collar | cnc | FBgn0000338 | Mohler et al. 1995 | this study |  |
| caudal | cad | FBgn0000251 | Macdonald and Struhl 1986 | ND | Rajewsky et al. 2002 |
| Deformed | Dfd | FBgn0000439 | Martinez-Arias et al. 1987 | Regulski et al. 1991 |  |
| Dichaete | D | FBgn0000411 | Nambu and Nambu 1996; Russell et al. 1996 | ND |  |
| D-Stat | Stat92E | FBgn0016917 | Yan et al. 1996 | ND | Yan et al. 1996 |
| empty spiracles | ems | FBgn0000576 | Dalton et al. 1989; Walldorf and Gehring 1992 | Hartmann et al. 2001 |  |
| engrailed | en | FBgn0000577 | DiNardo et al. 1985; Weir and Kornberg 1985 | Florence et al. 1997 |  |
| even skipped | eve | FBgn0000606 | Frasch and Levine 1987; Macdonald et al. 1986 | Fujioka et al. 1999; Small et al. 1993; Small et al. 1992; Small et al. 1991 | Hoey et al. 1988 |
| fork head | fkh | FBgn0000659 | Weigel et al. 1989 | ND |  |
| frizzled | fz | FBgn0001085 | Bhanot et al. 1999; Chen and Struhl 1999; Muller et al. 1999 |  |  |
| frizzled 2 | fz2 | FBgn0016797 | Bhanot et al. 1996 |  |  |
| ftz-f1 | ftz-f1 | FBgn0001078 | Florence et al. 1997; Yu et al. 1997 | ND | Florence et al. 1997; Ueda et al. 1990; Yu et al. 1997 |
| fushi tarazu | ftz | FBgn0001077 | Hafen et al. 1984 | Hiromi et al. 1985; Pick et al. 1990 | Papatsenko et al. 2002 |
| giant | gt | FBgn0001150 | Mohler et al. 1989 | this study; Berman et al. 2002 | Capovilla et al. 1992  Shimell et al. 2000 |
| gooseberry | gsb | FBgn0001148 | Bopp et al. 1986; Bopp et al. 1989 | Bouchard et al. 2000 |  |
| Goosecoid | Gsc | FBgn0010323 | Goriely et al. 1996 | ND |  |
| hairy | h | FBgn0001168 | Ingham et al. 1985 | Howard and Struhl 1990; Langeland and Carroll 1993; Riddihough and Ish-Horowicz 1991 | Van Doren et al. 1994 |
| hedgehog | hh | FBgn0004644 | Lee et al. 1992; Tabata et al. 1992 |  |  |
| huckebein | hkb | FBgn0001204 | Bronner and Jackle 1991 | Hader et al. 2000 |  |
| hunchback | hb | FBgn0001180 | Tautz 1988 | Schroder et al. 1988 | Rajewsky et al. 2002 |
| invected | inv | FBgn0001269 | Coleman et al. 1987 |  |  |
| ken and barbie | ken | FBgn0011236 | Kuhnlein et al. 1998 | ND |  |
| knirps | kni | FBgn0001320 | Nauber et al. 1988 | Pankratz et al. 1992; Rivera-Pomar et al. 1995; this study | Rajewsky et al. 2002 |
| knirps-like | knrl | FBgn0001323 | Rothe et al. 1989 | this study |  |
| Kruppel | Kr | FBgn0001325 | Gaul and Jackle 1987; Knipple et al. 1985 | Hoch et al. 1990; Hoch et al. 1991 | Rajewsky et al. 2002 |
| labial | lab | FBgn0002522 | Diederich et al. 1989; Mlodzik et al. 1988 |  |  |
| no ocelli | noc | FBgn0005771 | Cheah et al. 1994 | ND |  |
| nubbin | nub | FBgn0002970 | Cockerill et al. 1993; Lloyd and Sakonju 1991 | this study | Verrijzer et al. 1992 |
| ocelliless | oc | FBgn0004102 | Finkelstein et al. 1990 | Gao and Finkelstein 1998 |  |
| odd paired | opa | FBgn0003002 | Benedyk et al. 1994 | ND |  |
| odd skipped | odd | FBgn0002985 | Coulter et al. 1990 | ND |  |
| Optix | Optix | FBgn0025360 | Seo et al. 1999 | ND |  |
| paired | prd | FBgn0003145 | Baumgartner and Noll 1990 | Gutjahr et al. 1994 | Papatsenko et al. 2002 |
| patched | ptc | FBgn0003892 | Hooper and Scott 1989; Nakano et al. 1989 |  |  |
| pdm2 | pdm2 | FBgn0004394 | Cockerill et al. 1993  Bhat and Schedl 1994 | this study | Verrijzer et al. 1992 |
| proboscipedia | pb | FBgn0051481 | Randazzo et al. 1991 |  |  |
| runt | run | FBgn0003300 | Gergen and Butler 1988 | Klingler et al. 1996 | Melnikova et al. 1993 |
| Sex combs reduced | Scr | FBgn0003339 | Martinez-Arias et al. 1987 |  |  |
| sloppy paired 1 | slp1 | FBgn0003430 | Grossniklaus et al. 1992 | Lee and Frasch 2000 |  |
| sloppy paired 2 | slp2 | FBgn0004567 | Grossniklaus et al. 1992 | this study |  |
| spalt major | salm | FBgn0004579 | Kuhnlein et al. 1994 | Kuhnlein et al. 1997 |  |
| tailless | tll | FBgn0003720 | Pignoni et al. 1990 | Liaw and Lengyel 1993; Liaw et al. 1993; Rudolph et al. 1997 | Rajewsky et al. 2002 |
| teashirt | tsh | FBgn0003866 | Roder et al. 1992 | Core et al. 1997 |  |
| torso | tor | FBgn0003733 | Casanova and Struhl 1989; Sprenger and Nusslein-Volhard 1992; Sprenger et al. 1989 | ND | Rajewsky et al. 2002 |
| tramtrack | ttk | FBgn0003870 | Brown and Wu 1993 | ND | Papatsenko et al. 2002 |
| Ultrabithorax | Ubx | FBgn0003944 | Beachy et al. 1985; Lipshitz et al. 1987 | Qian et al. 1993; Zhang et al. 1991 |  |
| wingless | wg | FBgn0004009 | Baker 1988; Rijsewijk et al. 1987 |  |  |

**References**

Baker NE (1988) Localization of transcripts from the wingless gene in whole Drosophila embryos. Development 103: 289-298.

Baumgartner S, Noll M (1990) Network of interactions among pair-rule genes regulating paired expression during primordial segmentation of Drosophila. Mech Dev 33: 1-18.

Beachy PA, Helfand SL, Hogness DS (1985) Segmental distribution of bithorax complex proteins during Drosophila development. Nature 313: 545-551.

Benedyk MJ, Mullen JR, DiNardo S (1994) odd-paired: a zinc finger pair-rule protein required for the timely activation of engrailed and wingless in Drosophila embryos. Genes Dev 8: 105-117.

Berman BP, Nibu Y, Pfeiffer BD, Tomancak P, Celniker SE et al. (2002) Exploiting transcription factor binding site clustering to identify cis- regulatory modules involved in pattern formation in the Drosophila genome. Proc Natl Acad Sci U S A 99: 757-762.

Bhanot P, Brink M, Samos CH, Hsieh JC, Wang Y et al. (1996) A new member of the frizzled family from Drosophila functions as a Wingless receptor. Nature 382: 225-230.

Bhanot P, Fish M, Jemison JA, Nusse R, Nathans J et al. (1999) Frizzled and Dfrizzled-2 function as redundant receptors for Wingless during Drosophila embryonic development. Development 126: 4175-4186.

Bhat KM, Schedl P (1994) The Drosophila miti-mere gene, a member of the POU family, is required for the specification of the RP2/sibling lineage during neurogenesis. Development 120: 1483-1501.

Bopp D, Burri M, Baumgartner S, Frigerio G, Noll M (1986) Conservation of a large protein domain in the segmentation gene paired and in functionally related genes of Drosophila. Cell 47: 1033-1040.

Bopp D, Jamet E, Baumgartner S, Burri M, Noll M (1989) Isolation of two tissue-specific Drosophila paired box genes, Pox meso and Pox neuro. Embo J 8: 3447-3457.

Bouchard M, St-Amand J, Cote S (2000) Combinatorial activity of pair-rule proteins on the Drosophila gooseberry early enhancer. Dev Biol 222: 135-146.

Bronner G, Jackle H (1991) Control and function of terminal gap gene activity in the posterior pole region of the Drosophila embryo. Mech Dev 35: 205-211.

Brown JL, Wu C (1993) Repression of Drosophila pair-rule segmentation genes by ectopic expression of tramtrack. Development 117: 45-58.

Capovilla M, Eldon ED, Pirrotta V (1992) The giant gene of Drosophila encodes a b-ZIP DNA-binding protein that regulates the expression of other segmentation gap genes. Development 114: 99-112.

Casanova J, Struhl G (1989) Localized surface activity of torso, a receptor tyrosine kinase, specifies terminal body pattern in Drosophila. Genes Dev 3: 2025-2038.

Celniker SE, Keelan DJ, Lewis EB (1989) The molecular genetics of the bithorax complex of Drosophila: characterization of the products of the Abdominal-B domain. Genes Dev 3: 1424-1436.

Cheah PY, Meng YB, Yang X, Kimbrell D, Ashburner M et al. (1994) The Drosophila l(2)35Ba/nocA gene encodes a putative Zn finger protein involved in the development of the embryonic brain and the adult ocellar structures. Mol Cell Biol 14: 1487-1499.

Chen CM, Struhl G (1999) Wingless transduction by the Frizzled and Frizzled2 proteins of Drosophila. Development 126: 5441-5452.

Cockerill KA, Billin AN, Poole SJ (1993) Regulation of expression domains and effects of ectopic expression reveal gap gene-like properties of the linked pdm genes of Drosophila. Mech Dev 41: 139-153.

Coleman KG, Poole SJ, Weir MP, Soeller WC, Kornberg T (1987) The invected gene of Drosophila: sequence analysis and expression studies reveal a close kinship to the engrailed gene. Genes Dev 1: 19-28.

Core N, Charroux B, McCormick A, Vola C, Fasano L et al. (1997) Transcriptional regulation of the Drosophila homeotic gene teashirt by the homeodomain protein Fushi tarazu. Mech Dev 68: 157-172.

Coulter DE, Swaykus EA, Beran-Koehn MA, Goldberg D, Wieschaus E et al. (1990) Molecular analysis of odd-skipped, a zinc finger encoding segmentation gene with a novel pair-rule expression pattern. Embo J 9: 3795-3804.

Dalton D, Chadwick R, McGinnis W (1989) Expression and embryonic function of empty spiracles: a Drosophila homeo box gene with two patterning functions on the anterior-posterior axis of the embryo. Genes Dev 3: 1940-1956.

Diederich RJ, Merrill VK, Pultz MA, Kaufman TC (1989) Isolation, structure, and expression of labial, a homeotic gene of the Antennapedia Complex involved in Drosophila head development. Genes Dev 3: 399-414.

DiNardo S, Kuner JM, Theis J, O'Farrell PH (1985) Development of embryonic pattern in D. melanogaster as revealed by accumulation of the nuclear engrailed protein. Cell 43: 59-69.

Driever W, Nusslein-Volhard C (1988) The bicoid protein determines position in the Drosophila embryo in a concentration-dependent manner. Cell 54: 95-104.

Driever W, Nusslein-Volhard C (1988) A gradient of bicoid protein in Drosophila embryos. Cell 54: 83-93.

Finkelstein R, Smouse D, Capaci TM, Spradling AC, Perrimon N (1990) The orthodenticle gene encodes a novel homeo domain protein involved in the development of the Drosophila nervous system and ocellar visual structures. Genes Dev 4: 1516-1527.

Florence B, Guichet A, Ephrussi A, Laughon A (1997) Ftz-F1 is a cofactor in Ftz activation of the Drosophila engrailed gene. Development 124: 839-847.

Frasch M, Levine M (1987) Complementary patterns of even-skipped and fushi tarazu expression involve their differential regulation by a common set of segmentation genes in Drosophila. Genes Dev 1: 981-995.

Fujioka M, Emi-Sarker Y, Yusibova GL, Goto T, Jaynes JB (1999) Analysis of an even-skipped rescue transgene reveals both composite and discrete neuronal and early blastoderm enhancers, and multi-stripe positioning by gap gene repressor gradients. Development 126: 2527-2538.

Gao Q, Finkelstein R (1998) Targeting gene expression to the head: the Drosophila orthodenticle gene is a direct target of the Bicoid morphogen. Development 125: 4185-4193.

Gaul U, Jackle H (1987) Pole region-dependent repression of the Drosophila gap gene Kruppel by maternal gene products. Cell 51: 549-555.

Gergen JP, Butler BA (1988) Isolation of the Drosophila segmentation gene runt and analysis of its expression during embryogenesis. Genes Dev 2: 1179-1193.

Goriely A, Stella M, Coffinier C, Kessler D, Mailhos C et al. (1996) A functional homologue of goosecoid in Drosophila. Development 122: 1641-1650.

Grossniklaus U, Pearson RK, Gehring WJ (1992) The Drosophila sloppy paired locus encodes two proteins involved in segmentation that show homology to mammalian transcription factors. Genes Dev 6: 1030-1051.

Gutjahr T, Vanario-Alonso CE, Pick L, Noll M (1994) Multiple regulatory elements direct the complex expression pattern of the Drosophila segmentation gene paired. Mech Dev 48: 119-128.

Hader T, Wainwright D, Shandala T, Saint R, Taubert H et al. (2000) Receptor tyrosine kinase signaling regulates different modes of Groucho- dependent control of Dorsal. Curr Biol 10: 51-54.

Hafen E, Kuroiwa A, Gehring WJ (1984) Spatial distribution of transcripts from the segmentation gene fushi tarazu during Drosophila embryonic development. Cell 37: 833-841.

Hartmann B, Reichert H, Walldorf U (2001) Interaction of gap genes in the Drosophila head: tailless regulates expression of empty spiracles in early embryonic patterning and brain development. Mech Dev 109: 161-172.

Hiromi Y, Kuroiwa A, Gehring WJ (1985) Control elements of the Drosophila segmentation gene fushi tarazu. Cell 43: 603-613.

Hoch M, Schroder C, Seifert E, Jackle H (1990) cis-acting control elements for Kruppel expression in the Drosophila embryo. Embo J 9: 2587-2595.

Hoch M, Seifert E, Jackle H (1991) Gene expression mediated by cis-acting sequences of the Kruppel gene in response to the Drosophila morphogens bicoid and hunchback. Embo J 10: 2267-2278.

Hoey T, Warrior R, Manak J, Levine M (1988) DNA-binding activities of the Drosophila melanogaster even-skipped protein are mediated by its homeo domain and influenced by protein context. Mol Cell Biol 8: 4598-4607.

Hooper JE, Scott MP (1989) The Drosophila patched gene encodes a putative membrane protein required for segmental patterning. Cell 59: 751-765.

Howard KR, Struhl G (1990) Decoding positional information: regulation of the pair-rule gene hairy. Development 110: 1223-1231.

Ingham P, Howard K, Ish-Horowicz D (1985) Transcription pattern of the Drosophila segmentation gene hairy. Nature 318: 493-445.

Karch F, Bender W, Weiffenbach B (1990) abdA expression in Drosophila embryos. Genes Dev 4: 1573-1587.

Klingler M, Soong J, Butler B, Gergen JP (1996) Disperse versus compact elements for the regulation of runt stripes in Drosophila. Dev Biol 177: 73-84.

Knipple DC, Seifert E, Rosenberg UB, Preiss A, Jackle H (1985) Spatial and temporal patterns of Kruppel gene expression in early Drosophila embryos. Nature 317: 40-44.

Kuhnlein RP, Bronner G, Taubert H, Schuh R (1997) Regulation of Drosophila spalt gene expression. Mech Dev 66: 107-118.

Kuhnlein RP, Chen CK, Schuh R (1998) A transcription unit at the ken and barbie gene locus encodes a novel Drosophila zinc finger protein. Mech Dev 79: 161-164.

Kuhnlein RP, Frommer G, Friedrich M, Gonzalez-Gaitan M, Weber A et al. (1994) spalt encodes an evolutionarily conserved zinc finger protein of novel structure which provides homeotic gene function in the head and tail region of the Drosophila embryo. Embo J 13: 168-179.

Langeland JA, Carroll SB (1993) Conservation of regulatory elements controlling hairy pair-rule stripe formation. Development 117: 585-596.

Lee HH, Frasch M (2000) Wingless effects mesoderm patterning and ectoderm segmentation events via induction of its downstream target sloppy paired. Development 127: 5497-5508.

Lee JJ, von Kessler DP, Parks S, Beachy PA (1992) Secretion and localized transcription suggest a role in positional signaling for products of the segmentation gene hedgehog. Cell 71: 33-50.

Levine M, Hafen E, Garber RL, Gehring WJ (1983) Spatial distribution of Antennapedia transcripts during Drosophila development. Embo J 2: 2037-2046.

Liaw GJ, Lengyel JA (1993) Control of tailless expression by bicoid, dorsal and synergistically interacting terminal system regulatory elements. Mech Dev 40: 47-61.

Liaw GJ, Steingrimsson E, Pignoni F, Courey AJ, Lengyel JA (1993) Characterization of downstream elements in a Raf-1 pathway. Proc Natl Acad Sci U S A 90: 858-862.

Lipshitz HD, Peattie DA, Hogness DS (1987) Novel transcripts from the Ultrabithorax domain of the bithorax complex. Genes Dev 1: 307-322.

Lloyd A, Sakonju S (1991) Characterization of two Drosophila POU domain genes, related to oct-1 and oct-2, and the regulation of their expression patterns. Mech Dev 36: 87-102.

Macdonald PM, Ingham P, Struhl G (1986) Isolation, structure, and expression of even-skipped: a second pair- rule gene of Drosophila containing a homeo box. Cell 47: 721-734.

Macdonald PM, Struhl G (1986) A molecular gradient in early Drosophila embryos and its role in specifying the body pattern. Nature 324: 537-545.

Martinez-Arias A, Ingham PW, Scott MP, Akam ME (1987) The spatial and temporal deployment of Dfd and Scr transcripts throughout development of Drosophila. Development 100: 673-683.

Melnikova IN, Crute BE, Wang S, Speck NA (1993) Sequence specificity of the core-binding factor. J Virol 67: 2408-2411.

Mlodzik M, Fjose A, Gehring WJ (1988) Molecular structure and spatial expression of a homeobox gene from the labial region of the Antennapedia-complex. Embo J 7: 2569-2578.

Mohler J, Eldon ED, Pirrotta V (1989) A novel spatial transcription pattern associated with the segmentation gene, giant, of Drosophila. Embo J 8: 1539-1548.

Mohler J, Mahaffey JW, Deutsch E, Vani K (1995) Control of Drosophila head segment identity by the bZIP homeotic gene cnc. Development 121: 237-247.

Muller H, Samanta R, Wieschaus E (1999) Wingless signaling in the Drosophila embryo: zygotic requirements and the role of the frizzled genes. Development 126: 577-586.

Nakano Y, Guerrero I, Hidalgo A, Taylor A, Whittle JR et al. (1989) A protein with several possible membrane-spanning domains encoded by the Drosophila segment polarity gene patched. Nature 341: 508-513.

Nambu PA, Nambu JR (1996) The Drosophila fish-hook gene encodes a HMG domain protein essential for segmentation and CNS development. Development 122: 3467-3475.

Nauber U, Pankratz MJ, Kienlin A, Seifert E, Klemm U et al. (1988) Abdominal segmentation of the Drosophila embryo requires a hormone receptor-like protein encoded by the gap gene knirps. Nature 336: 489-492.

Pankratz MJ, Busch M, Hoch M, Seifert E, Jackle H (1992) Spatial control of the gap gene knirps in the Drosophila embryo by posterior morphogen system. Science 255: 986-989.

Papatsenko DA, Makeev VJ, Lifanov AP, Regnier M, Nazina AG et al. (2002) Extraction of functional binding sites from unique regulatory regions: the Drosophila early developmental enhancers. Genome Res 12: 470-481.

Pick L, Schier A, Affolter M, Schmidt-Glenewinkel T, Gehring WJ (1990) Analysis of the ftz upstream element: germ layer-specific enhancers are independently autoregulated. Genes Dev 4: 1224-1239.

Pignoni F, Baldarelli RM, Steingrimsson E, Diaz RJ, Patapoutian A et al. (1990) The Drosophila gene tailless is expressed at the embryonic termini and is a member of the steroid receptor superfamily. Cell 62: 151-163.

Qian S, Capovilla M, Pirrotta V (1993) Molecular mechanisms of pattern formation by the BRE enhancer of the Ubx gene. Embo J 12: 3865-3877.

Rajewsky N, Vergassola M, Gaul U, Siggia ED (2002) Computational detection of genomic cis-regulatory modules applied to body patterning in the early Drosophila embryo. BMC Bioinformatics 3: 30.

Randazzo FM, Cribbs DL, Kaufman TC (1991) Rescue and regulation of proboscipedia: a homeotic gene of the Antennapedia Complex. Development 113: 257-271.

Regulski M, Dessain S, McGinnis N, McGinnis W (1991) High-affinity binding sites for the Deformed protein are required for the function of an autoregulatory enhancer of the Deformed gene. Genes Dev 5: 278-286.

Riddihough G, Ish-Horowicz D (1991) Individual stripe regulatory elements in the Drosophila hairy promoter respond to maternal, gap, and pair-rule genes. Genes Dev 5: 840-854.

Rijsewijk F, Schuermann M, Wagenaar E, Parren P, Weigel D et al. (1987) The Drosophila homolog of the mouse mammary oncogene int-1 is identical to the segment polarity gene wingless. Cell 50: 649-657.

Rivera-Pomar R, Lu X, Perrimon N, Taubert H, Jackle H (1995) Activation of posterior gap gene expression in the Drosophila blastoderm. Nature 376: 253-256.

Roder L, Vola C, Kerridge S (1992) The role of the teashirt gene in trunk segmental identity in Drosophila. Development 115: 1017-1033.

Rothe M, Nauber U, Jackle H (1989) Three hormone receptor-like Drosophila genes encode an identical DNA- binding finger. Embo J 8: 3087-3094.

Rudolph KM, Liaw GJ, Daniel A, Green P, Courey AJ et al. (1997) Complex regulatory region mediating tailless expression in early embryonic patterning and brain development. Development 124: 4297-4308.

Russell SR, Sanchez-Soriano N, Wright CR, Ashburner M (1996) The Dichaete gene of Drosophila melanogaster encodes a SOX-domain protein required for embryonic segmentation. Development 122: 3669-3676.

Schroder C, Tautz D, Seifert E, Jackle H (1988) Differential regulation of the two transcripts from the Drosophila gap segmentation gene hunchback. Embo J 7: 2881-2887.

Seo HC, Curtiss J, Mlodzik M, Fjose A (1999) Six class homeobox genes in drosophila belong to three distinct families and are involved in head development. Mech Dev 83: 127-139.

Shimell MJ, Peterson AJ, Burr J, Simon JA, O'Connor MB (2000) Functional analysis of repressor binding sites in the iab-2 regulatory region of the abdominal-A homeotic gene. Dev Biol 218: 38-52.

Small S, Arnosti DN, Levine M (1993) Spacing ensures autonomous expression of different stripe enhancers in the even-skipped promoter. Development 119: 762-772.

Small S, Blair A, Levine M (1992) Regulation of even-skipped stripe 2 in the Drosophila embryo. Embo J 11: 4047-4057.

Small S, Kraut R, Hoey T, Warrior R, Levine M (1991) Transcriptional regulation of a pair-rule stripe in Drosophila. Genes Dev 5: 827-839.

Sprenger F, Nusslein-Volhard C (1992) Torso receptor activity is regulated by a diffusible ligand produced at the extracellular terminal regions of the Drosophila egg. Cell 71: 987-1001.

Sprenger F, Stevens LM, Nusslein-Volhard C (1989) The Drosophila gene torso encodes a putative receptor tyrosine kinase. Nature 338: 478-483.

Tabata T, Eaton S, Kornberg TB (1992) The Drosophila hedgehog gene is expressed specifically in posterior compartment cells and is a target of engrailed regulation. Genes Dev 6: 2635-2645.

Tautz D (1988) Regulation of the Drosophila segmentation gene hunchback by two maternal morphogenetic centres. Nature 332: 281-284.

Ueda H, Sonoda S, Brown JL, Scott MP, Wu C (1990) A sequence-specific DNA-binding protein that activates fushi tarazu segmentation gene expression. Genes Dev 4: 624-635.

Van Doren M, Bailey AM, Esnayra J, Ede K, Posakony JW (1994) Negative regulation of proneural gene activity: hairy is a direct transcriptional repressor of achaete. Genes Dev 8: 2729-2742.

Verrijzer CP, Alkema MJ, van Weperen WW, Van Leeuwen HC, Strating MJ et al. (1992) The DNA binding specificity of the bipartite POU domain and its subdomains. Embo J 11: 4993-5003.

Walldorf U, Gehring WJ (1992) Empty spiracles, a gap gene containing a homeobox involved in Drosophila head development. Embo J 11: 2247-2259.

Weigel D, Jurgens G, Kuttner F, Seifert E, Jackle H (1989) The homeotic gene fork head encodes a nuclear protein and is expressed in the terminal regions of the Drosophila embryo. Cell 57: 645-658.

Weir MP, Kornberg T (1985) Patterns of engrailed and fushi tarazu transcripts reveal novel intermediate stages in Drosophila segmentation. Nature 318: 433-439.

Wimmer EA, Jackle H, Pfeifle C, Cohen SM (1993) A Drosophila homologue of human Sp1 is a head-specific segmentation gene. Nature 366: 690-694.

Wimmer EA, Simpson-Brose M, Cohen SM, Desplan C, Jackle H (1995) Trans- and cis-acting requirements for blastodermal expression of the head gap gene buttonhead. Mech Dev 53: 235-245.

Yan R, Small S, Desplan C, Dearolf CR, Darnell JE, Jr. (1996) Identification of a Stat gene that functions in Drosophila development. Cell 84: 421-430.

Yu Y, Li W, Su K, Yussa M, Han W et al. (1997) The nuclear hormone receptor Ftz-F1 is a cofactor for the Drosophila homeodomain protein Ftz. Nature 385: 552-555.

Zhang CC, Muller J, Hoch M, Jackle H, Bienz M (1991) Target sequences for hunchback in a control region conferring Ultrabithorax expression boundaries. Development 113: 1171-1179.
